# Sodium and Potassium Intake in Healthy Adults in Thessaloniki Greater Metropolitan Area—The Salt Intake in Northern Greece (SING) Study

**DOI:** 10.3390/nu9040417

**Published:** 2017-04-22

**Authors:** Eleni Vasara, Georgios Marakis, Joao Breda, Petros Skepastianos, Maria Hassapidou, Anthony Kafatos, Nikolaos Rodopaios, Alexandra A. Koulouri, Francesco P. Cappuccio

**Affiliations:** 1 Laboratory of Animal Physiology, Department of Zoology, School of Biology, Aristotle University of Thessaloniki, Thessaloniki 54124, Greece; evasara@bio.auth.gr; 2 Nutrition Policy and Research Directorate, Hellenic Food Authority, 124 Kifisias Av. & 2 Iatridou Str., Athens 11526, Greece; gmarakis@efet.gr; 3 Division of Noncommunicable Diseases and Promoting Health through the Life-Course, WHO Regional Office for Europe, Copenhagen DK-2100, Denmark; rodriguesdasilvabred@who.int; 4 Department of Medical Laboratory Studies, Alexander Technological and Educational Institute of Thessaloniki, Sindos, Thessaloniki 57400, Greece; pskep@otenet.gr; 5 Department of Nutrition and Dietetics, Alexander Technological and Educational Institute of Thessaloniki, Sindos, Thessaloniki 57400, Greece; mnhas@gmail.com; 6 Department of Social Medicine, Preventive Medicine and Nutrition Clinic, Medical School, University of Crete, Heraklion 71003, Crete, Greece; kafatos@med.uoc.gr (A.K.); nikow1966@yahoo.gr (N.R.); alexkoulou@yahoo.com (A.A.K.); 7 Division of Health Sciences (Mental Health & Wellbeing), Warwick Medical School, University of Warwick, Coventry CV4 7AL, UK; 8 University Hospitals Coventry & Warwickshire NHS Trust, Coventry, CV2 2DX, UK

**Keywords:** salt, sodium, potassium, intake, MedDietScore, Greece

## Abstract

A reduction in population sodium (as salt) consumption is a global health priority, as well as one of the most cost-effective strategies to reduce the burden of cardiovascular disease. High potassium intake is also recommended to reduce cardiovascular disease. To establish effective policies for setting targets and monitoring effectiveness within each country, the current level of consumption should be known. Greece lacks data on actual sodium and potassium intake. The aim of the present study was therefore to assess dietary salt (using sodium as biomarker) and potassium intakes in a sample of healthy adults in northern Greece, and to determine whether adherence to a Mediterranean diet is related to different sodium intakes or sodium-to-potassium ratio. A cross-sectional survey was carried out in the Thessaloniki greater metropolitan area (northern Greece) (*n* = 252, aged 18–75 years, 45.2% males). Participants’ dietary sodium and potassium intakes were determined by 24-hour urinary sodium and potassium excretions. In addition, we estimated their adherence to Mediterranean diet by the use of an 11-item MedDietScore (range 0–55). The mean sodium excretion was 175 (SD 72) mmol/day, equivalent to 4220 (1745) mg of sodium or 10.7 (4.4) g of salt per day, and the potassium excretion was 65 (25) mmol/day, equivalent to 3303 (1247) mg per day. Men had higher sodium and potassium excretions compared to women. Only 5.6% of the sample had salt intake <5 g/day, which is the target intake recommended by the World Health Organization. Mean sodium-to-potassium excretion ratio was 2.82 (1.07). There was no significant difference in salt or potassium intake or their ratio across MedDietScore quartiles. No significant relationships were found between salt intake and adherence to a Mediterranean diet, suggesting that the perception of the health benefits of the Mediterranean diet does not hold when referring to salt consumption. These results suggest the need for a larger, nation-wide survey on salt intake in Greece and underline the importance of continuation of salt reduction initiatives in Greece.

## 1. Introduction

Non-communicable diseases are the leading causes of death in Greece and worldwide. High blood pressure and unhealthy diet are among the risk factors that account for most of the disease burden in Greece [1]. Specifically, according to the most recent nation-wide health and diet survey in Greece, four out of ten adults have raised blood pressure [2] (p. 55). There is compelling evidence from experimental, epidemiological, migration and intervention studies as well as meta-analyses that high salt intake is associated with raised blood pressure and adverse cardiovascular health (i.e., coronary heart disease and stroke) (e.g., [3,4,5,6]), despite the publication of a small number of controversial scientific papers using flawed methodologies [7,8]. In addition, high salt intake is related to adverse health effects independent of its effects on blood pressure [9].

The World Health Organization currently recommends that adults should consume no more than 5 g of salt daily [10]. Even though sodium intake varies in populations around the world, in the vast majority of populations, salt intake is high and it exceeds both physiologic requirements and recommendations [11,12]. Greece seems to lack data on actual salt intake [13]. Salt reduction strategies in the European Union, including Greece, encompass monitoring and evaluation actions as one of their important pillars. Hence, comprehensive, current data on salt intake in Greece are urgently needed, using at least one accurately collected 24-hour urine sample for assessing sodium excretion, which is regarded as the gold standard method to assess salt consumption, at least for a population average [14,15].

In contrast to sodium, evidence from epidemiologic studies and randomized trials point to the beneficial effects of dietary potassium on blood pressure and cardiovascular health [16,17,18]. This effect is more pronounced in those with high sodium intake [19]. It has been suggested that individuals with diets that are low in potassium are particularly vulnerable to the hypertensive effects of high sodium intake [20,21]. Hence, the ratio of sodium-to-potassium may be more reliable than either nutrient alone in predicting the risk of cardiovascular disease [22,23]. In addition to sodium, potassium can also be determined accurately in 24-hour urine collections, hence avoiding the need to rely on reported dietary intake data and national up-to-date food composition tables.

The primary objective of the present study was to estimate the average population sodium and potassium intakes in northern Greece. The study also aimed to investigate whether adherence to a Mediterranean diet is related to different salt intakes or the sodium-to-potassium ratio.

## 2. Methods

### 2.1. Participants and Recruitment

Two hundred and seventy-four men and women (aged 18–75 years) participated in the Salt Intake in Northern Greece (SING) study. The investigation took place in northern Greece, mostly in the Thessaloniki greater metropolitan area (the second largest city in Greece). Recruitment was done at various sites and venues including churches and workplaces, based on an opportunistic sampling [15]. This approach has been recently shown to be suitable and free from significant bias when assessing population group average salt consumption [15]. Efforts were taken to avoid recruiting individuals who were particularly worried about their salt intake or their blood pressure and who might, as a consequence, have altered their diet. In order to attain that, adults were initially invited to participate in a nutrition survey, without specifying which nutrients would be investigated or how their intake would be assessed. Once people expressed interest for participating in a nutrition survey, a quick screening took place in order to exclude those who met the exclusion criteria. Pregnant and lactating women were excluded from the study. Other exclusions were those with a medical diagnosis of hypertension (whether on an anti-hypertensive treatment or not), diabetes mellitus as well as those with heart, liver, renal, gastrointestinal or neoplastic diseases. 

Eligible volunteers were then told how the study would be conducted and what would be required during their participation. Detailed written and verbal instructions were given to eligible volunteers, before receiving their informed consent to participate. It was carefully explained to participants how to collect their urine for 24 h, emphasizing the importance of providing a complete collection. In an effort to minimise conscious or unconscious modification of their diet or dietary practices (e.g., avoiding adding salt on the plate or avoiding high salt foods), participants were told that the aim of the 24-hour urine collection was to investigate the dietary intake of some nutrients, without specifying that the nutrients of this investigation were sodium and potassium. Participants were also requested not to change their diet before or during the day of the urine collection (e.g., skip a major meal that they normally have or follow a special diet that day).

Sample recruitment and urine collection were confined to one calendar year, commencing in February 2015 and completed in March 2016. No urinary samples were collected during festive seasons. No financial incentive was offered to participants. In order to motivate individuals to participate in this study, it was specified that participants would be notified of their own results as well as the general outcomes of the study. The study was approved by the Ethics Committee of the Alexander Technological and Educational Institute of Thessaloniki, and participants provided written informed consent to take part. 

### 2.2. Data Collection

Height and weight were measured in subjects wearing light clothing, without shoes, using standardized equipment. Body weight was recorded using a Tanita BWB-800S digital scale (Tanita Europe BV, Amsterdam, The Netherlands) to the nearest 0.1 Kg and body height was measured using a stable stadiometer to the nearest 0.1 cm. Body mass index was calculated as weight (Kg)/height (m^2^). Waist circumference (in cm) was measured around the midpoint between the costal margin and the iliac crest during expiration. Blood pressure was measured in triplicate, after a 10 min rest, using fully automatic Omron blood pressure monitor (Omron RX Classic II, Kyoto, Japan). The first reading was discarded and the mean of the second and third readings was calculated.

A single 24-hour urine collection was obtained from the participants. The first void upon waking on the day of collection was discarded. Participants then collected all voided urine up to, and including, the first void the following morning in multiple 500 mL screw-cup bottles. The exact times at the beginning and the end of urine collection were noted by the participants. The urine volume of the 24-hour collection was measured in the lab and a 10 mL aliquot was stored at −20 °C until analysis. Urinary sodium and potassium excretions were determined by ion-selective electrode potentiometry (ATVIA 1800 Siemens, ISE buffer Siemens AG, Munich, Germany) and by taking into account the exact 24-hour adjusted urinary volume. The sodium to potassium ratio in the 24-hour urine samples was also calculated. 

Urine collections were rejected if the participant admitted that a sample was missed from the collection or if the timing of the collection fell outside the range of 23–25 h. Urine collections were suspected to be inaccurate if urinary volumes were <500 mL. Para-aminobenzoic acid (PABA) marker was not used in this study. Despite its limitation, 24-hour urinary creatinine was used as a means to exclude urine collections judged to be incomplete. Creatinine was measured using the Jaffe method (ATVIA 1800 Siemens AG, Munich, Germany) [24]. If urinary creatinine (UCr) was less than 2 standard deviations from the mean, subjects were excluded from the statistical analysis.

For each individual, the 24-hour sodium or potassium excretion value (mEq/day or mmol/day) was calculated as the concentration of sodium or potassium in the urine (mmol/L) multiplied by the urinary volume (L/day). In order to convert urinary output to dietary intake, the urinary excretion of sodium or potassium values (mEq/day) were first converted to mg/day. Then, sodium values were multiplied by 1.05 (since urine output reflects approximately 95% of intake), while potassium values were multiplied by 1.3 [25]. The conversion from dietary sodium (Na) intake to salt (NaCl) intake was made by multiplying the sodium value by 2.542 (NaCl (g) = Na (g) × 2.542).

### 2.3. Adherence to Mediterranean Diet

The MedDietScore (MDS) was calculated for each participant to evaluate their adherence to the Mediterranean dietary pattern. The MedDietScore has previously been validated [26] and includes 11 main components. Specifically, it takes into account the frequency of consumption of nine food groups (i.e., servings/week for non-refined cereals, fruits, vegetables, potatoes, legumes, fish, red meat, poultry and full fat dairy products) as well as the frequency of consumption for olive oil (times/week) and alcohol (mL/day). Based on the recommended intake, monotonic ratings (with the exception of alcohol intake) were used in order to score the frequency of consumption of these foods. Individual ratings from 0 to 5 or the reverse were assigned for each of the above food groups/items, according to their position in the Mediterranean diet pyramid. The score ranges from 0 to 55, with higher values indicating greater adherence to the Mediterranean diet. 

### 2.4. Statistical Analysis

In general, to detect approximately 1 g reduction in salt intake over time using 24-hour urinary sodium excretion, with an estimated standard deviation of 75 mmol/day (α = 0.05, power = 0.80), a minimum sample of 120 individuals per stratum is recommended [27]. Hence, a minimum sample of 240 men and women participants was expected. Baseline sample characteristics (i.e., age, weight, height, Body Mass Index (BMI), waist circumference, blood pressure and MedDietScore) as well as salt intake, sodium and potassium excretion and intake values and their ratios are presented as mean (standard deviation). Age distribution, level of education and self-assessment of personal diet quality are presented as percentages.

Differences between groups were assessed using independent sample *t*-tests. Differences in sodium intake, potassium intake and sodium-to-potassium ratio across MedDietScore quartiles were assessed by one-way ANOVA. Pearson chi-square test was used to test the association between categorical variables. For all comparisons, significance level was at 5%. Statistical analysis was performed with SPSS statistical software package version 20, IBM (SPSS Inc., Chicago, IL, USA).

## 3. Results

### 3.1. Characteristics of Participants

After the initial screening, 274 volunteers gave written consent and provided a 24-hour urine collection. Eight participants were excluded because they admitted that one or more voids were lost or because their urine collection fell outside the range of 23–25 h. Six more were excluded on the grounds of their urinary volume being less than 500 mL per day, despite admitting that no urine was lost. Another six subjects were excluded because their 24-hour urinary creatinine excretion was more than two standard deviations from the mean. One individual was excluded because of low 24-hour urinary creatinine excretion and low urinary volume in spite of high weight, indicating possible under-collection, and finally one was excluded because of low 24-hour urinary creatinine excretion, but very high volume (>two standard deviations) suggesting possible over-dilution. Therefore, 22 participants in total were excluded from the analyses. The final sample comprised of 252 participants (92% of the initial sample) between 18 and 75 years old, of whom 45.2% were men and 54.8% were women.

The characteristics of the participants are shown in Table 1. There was no statistically significant difference in the mean age (*p* = 0.701), mean BMI (*p* = 0.234) and mean urine volume (*p* = 0.754) between male and female participants. Men had higher urinary creatinine excretion compared to women (*p* < 0.0001).

### 3.2. Sodium and Potassium Intakes

There was a considerable variation in sodium and potassium excretion. Average sodium and potassium excretions were higher in men than in women (Table 2). In men, daily sodium intake ranged from 797 mg to 11,213 mg, while in women dietary sodium ranged from 845 mg to 8489 mg. As far as potassium is concerned, its daily intake ranged from 1221 mg to 9001 mg in men and from 830 mg to 8044 mg in women. Men had significantly higher potassium intake compared to women (*p* = 0.001). The mean salt intake in the SING study was 10.7 (4.4) g/day (Table 2). Men had significantly higher salt intake (11.9 (4.7) g/day) compared to women (9.7 (3.9) g/day) (*p* < 0.0001).

**Figure 1:**
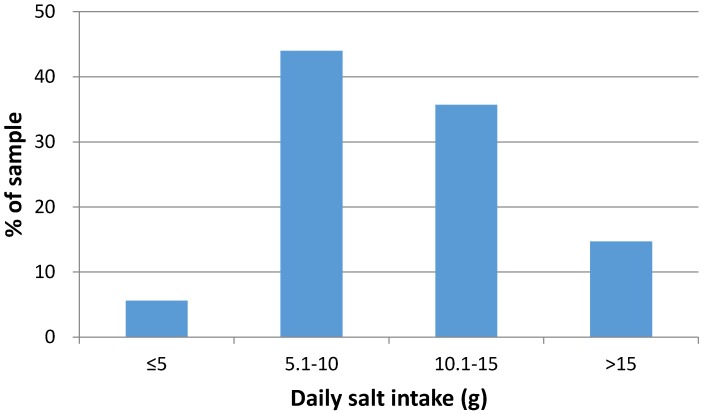
Distribution of single 24-hour salt intake estimates (see text for conversion of urinary excretions to estimates intakes).

With regard to the frequency distribution of salt data, only 5.6% of the sample (1.98% men and 3.57% women) had salt intake <5 g per day, which is the target intake recommended by the World Health Organization (WHO) guidelines (Figure 1). Moreover, since the dispersion in our sample (based on person-days of exposure) was substantially greater than the dispersion in a corresponding sample of usual intakes of individuals (due to the subtraction of intra-individual variance), the proportion of “persons” with an intake <5 g per day would be substantially less. In contrast, 50.4% of the study sample had a daily salt intake that exceeded 10 g per day. In a small percentage of participants (3.97%), salt intake exceeded 20 g per day. As far as potassium is concerned, 33.4% of participants had intakes equal or higher than the WHO recommendation of 3510 mg/day.

### 3.3. Sodium-to-Potassium Ratio

**Figure 2:**
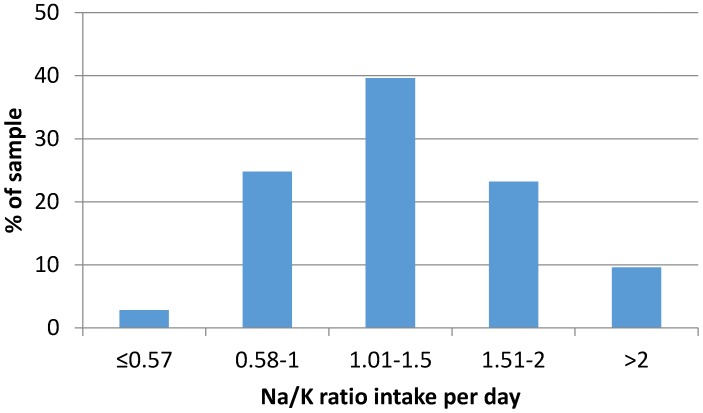
Distribution of dietary sodium-to-potassium ratios (mg/mg) in the sample of 24-hour intake estimates (see text for conversion of urinary excretions to estimates intakes).

The urinary sodium-to-potassium ratio was 2.82 (1.07) (Table 2). The dietary sodium-to-potassium intake ratio in the whole group was 1.34 (0.51). In the lowest salt intake quartile, the ratio was 0.98 (0.36), which rose to 1.70 (0.49) in the highest salt intake quartile, a statistically significant difference (*p* < 0.0001) (Figure 2). There was no statistically significant difference in dietary sodium-to-potassium ratio between genders (*p* = 0.478).

For only 2.8% of the sample of 24-hour measurements (1.2% men and 1.6% women) was the dietary sodium-to-potassium intake ratio (mg/mg) less than 0.57 (Figure 2). This chosen cut-off value results from the WHO guidelines on sodium and potassium for adults (i.e., 2000 mg Na/3510 mg K = 0.57). 

Finally, we did not detect seasonal variations in estimates of sodium and potassium excretion and dietary consumption between Spring-Summer and Autumn-Winter. 

### 3.4. Salt Intake and Adherence to the Mediterranean Diet

The mean MedDietScore of the sample was 30.5 (5.1), ranging from 14 to 45, with women having a statistically significant lower score compared to men (*p* < 0.0001) (Table 1). There was no statistically significant difference of the MedDietScore between the lowest (29.5 (5.1)) and highest (30.9 (5.1)) quartiles of salt intake (*p* = 0.124). Sodium intake, potassium intake and the sodium-to-potassium ratio by MedDietScore quartiles are shown in Table 3. There were no significant differences in sodium or potassium intake or their ratio across MedDietScore quartiles.

## 4. Discussion

This is the first study in northern Greece that estimates salt intake in a group of free-living healthy adults using 24-hour urinary excretion, which is the preferred method of obtaining data on salt intake in population surveys [12]. Furthermore, rigid controls were applied to exclude participants who were suspected of providing a problematic urine collection. While recruitment was done on an opportunistic basis, a recent study carried out in Australia has shown that group estimates of salt intake from such samples are not significantly different from those obtained from “random” samples [15]. If applicable to Greece, this would suggest that the average estimate of salt consumption is unlikely to be biased. In Greece, there are no nationally-specific guidelines or targets regarding sodium and potassium intakes other than those issued by WHO. Salt intake was, on average, double the current WHO recommendations. Less than 5% of the sample will have had usual intakes below the 5 g per day recommended limit, while one third met the current WHO recommendations of 3510 mg per day for potassium [28]. High salt intake was anticipated in this sample, since salt consumption in neighbouring countries with similar dietary habits is also high. For example, in Turkey, the average salt intake is about 15 g per day [29], while in Italy it is approximately 9 g per day [25].

The greater sodium and potassium intakes seen in men compared to women are in line with other studies [11,15,25,30] and may not only reflect differences in food choices but most probably differences in total food consumption, since men have greater energy requirements than women. The higher the body mass index, the greater the salt intake usually is. The mean body mass index of the participants in this study was in the “overweight” range, which is comparable to the mean body mass index of the Greek adult population as a whole, as reported in the first national health and diet survey [2].

It has been suggested that potassium intake should be at a level which will keep the urinary sodium-to-potassium ratio close to 1.0 (mmol/mmol) [31] or the dietary ratio close to 0.57 (mg/mg) to improve blood pressure. However, 97.2% of the sample had a dietary sodium-to-potassium intake ratio (mg/mg) above 0.57. If one considers the sodium-to-potassium excretion ratio, only two participants appeared to meet WHO recommendations (mg/mg). Whether using excretion or intake values, sodium-to-potassium ratio was high. These values are associated with poor cardiovascular outcomes [22,32]. Considering the differences in the ratio values obtained using sodium and potassium excretion from that using sodium and potassium intake, an agreement should be reached on which ratio to use for monitoring population progress [33].

Detailed information on food consumption was not collected and, as a result, the contribution of different dietary sources was not investigated. On average, men met WHO recommendations for potassium intake while women did not. Potassium is particularly abundant in fruits and vegetables, which are also part of the Mediterranean diet model. The mean MedDietScore was 30.5, which is comparable to the scores published for Greek populations in other studies. In the ATTICA study, the mean score was 25.5 (2.9) for men and 27.2 (3.2) for women [26], while in the more recent MEDIS study, participants from the Mani region in Greece (a rural region which keeps old traditions) had a mean MedDietScore of 32 (4.0) [34]. However, in our study, those who appear to adhere better to a Mediterranean diet did not have different salt intake or sodium-to-potassium ratio compared to those who adhere less to a Mediterranean diet. The addition of salt to salads and cooked vegetables as well as the high salt content of some traditional Greek foods, such as cheeses, pies and spreads [35], might account for this lack of association. Therefore, while sustained efforts to promote the traditional Mediterranean model of diet in Greece are important and necessary, these should also be accompanied with specific actions to reduce salt.

### Limitations of the Study

Two aspects of the study need further discussion. First, the use of an opportunistic sampling frame may introduce a bias in the overall estimate of salt consumption, affecting the validity of the survey. 24-hour urine collections often are a burden to participants of large population-based dietary surveys so that, despite great efforts and resources, response rates are often low [15,30,36,37]. A recent study has compared the results of an opportunistically recruited volunteer population sample where a random sampling had yielded a 16% response rate [15]. The average estimates of salt intake were comparable, suggesting that such an approach may provide a reasonable estimate of population salt intake. In our study, every possible step was taken to minimize the chance of recruiting individuals who were particularly interested in their salt intake or their blood pressure, and who might unconsciously have modified their consumption of salt during the time of the survey. Still, the possibility of selection bias cannot be excluded, since those who participated expressed interest in taking part in a nutrition-related survey and as a consequence may generally be more cautious about their diet. Similar concerns, however, could be raised for nutrition surveys which do not provide financial or other non-nutrition-related incentives. In addition, although a 24-hour period is necessary to capture the marked diurnal variation in sodium and water excretion, there is day-to-day variation in salt consumption (due to daily variations in salt intake as well as a possible infradian rhythmical variability) [38,39,40]. The high intra-individual variability, compared to the between-subjects variability, limits the ability to characterize individuals’ sodium excretion (i.e., salt intake). However, it does not much limit the ability to identify the average salt intake of groups (like Greek men and women, collectively) to support the valid evaluation of population intervention programmes over time. Finally, the survey was stopped in the months of July and August, to minimize the potential confounding effect of high temperature and excessive sweating. We feel that the estimates obtained in our study, whilst limited, provide enough evidence to support a national programme of population reduction in salt intake. 

The second limitation regards the study’s representativeness of the whole Greek population. The survey was performed in the urban and suburban areas of Thessaloniki, the largest city in northern Greece. Clearly, it is difficult to infer to the rest of the country. Greece has a widely spread territory, not only spanning from north to south but also with sharp contrasts between mountainous and sea areas and the myriad of islands. Their populations, whilst sharing some national traditions also reflected in common eating habits, do have distinctive local differences that might affect the amount of salt they usually consume. While limited information on dietary habits was obtained to estimate the MedDietScore, detailed long-term data on food consumption, through the use of a food diary or dietary recalls, were not obtained and, as a result, the main contributors to salt intake could not be assessed. Furthermore, university-educated participants were slightly overrepresented compared to the frequency of them in the national census of similar age. As salt consumption was lower in them, the group estimates obtained in our study may be a conservative estimate of an even higher intake in the general population.

## 5. Conclusions

Measurements of 24-hour urinary sodium and potassium excretion were carried out for the first time in a sample of healthy free-living adults in northern Greece. These measurements revealed that, in this population, salt consumption is high and above the WHO upper limit, whilst potassium consumption is still sub-optimal. No significant relationships were found between salt intake and adherence to a Mediterranean diet, suggesting that the perception of the health benefits of a Mediterranean diet does not hold when referring to salt consumption. These results should provide an impetus for public health authorities in Greece to continue their efforts towards meeting the WHO target of a 30% reduction in salt intake by 2025. In the absence of a more comprehensive national survey of habitual salt intake in Greece, our data provides a useful baseline against which to monitor the impact of future salt reduction initiatives.

## Figures and Tables

**Table 1:** Demographic data of the participants (*n* = 252).

	Total (*n* = 252)	Men (*n* = 114)	Women (*n* = 138)
Mean Age (years)	46.6 (16.6)	47.0 (16.2)	46.2 (17.0)
% in the range 18–34	26.8	27.4	27.0
% in the range 35–49	25.6	24.8	25.6
% in the range 50–64	35.6	36.3	35.0
% in the range 65–75	12.0	11.5	12.4
Height (cm)	169.3 (9.5)	176.8 (6.6)	163.1 (6.6) ****
Weight (kg)	77.0 (15.7)	85.2 (14.6)	70.3 (13.1) ****
BMI (kg/m^2^)	26.8 (4.7)	27.2 (4.1)	26.5 (5.1)
Waist circumference (cm)	87.6 (14.7)	94.8 (13.1)	81.6 (13.1) ****
Level of education (%)			
Non university graduates	60.3	51.8	67.4
University graduates	39.7	48.2	32.6 *
Self-assessment of personal diet quality (%)			
Good	61.3	62.0	60.7
Moderate	37.1	34.5	39.3
Bad	1.6	3.5	0
Systolic BP (mmHg)	126.5 (16.4)	129.9 (16.8)	123.7 (15.5) **
Diastolic BP (mmHg)	79.8 (11.9)	82.5 (12.5)	77.6 (10.9) ***
MedDietScore	30.5 (5.1)	31.8 (5.4)	29.4 (4.6) ****

BMI, Body Mass Index; BP, blood pressure. Results are presented as means (SD) or %. * *p* < 0.05; ** *p* ≤ 0.01; *** *p* ≤ 0.001; **** *p* ≤ 0.0001 vs. men.

**Table 2:** Mean sodium and potassium excretion, intakes and their ratio in men and women.

	Total (*n* = 252)	Men (*n* = 114)	Women (*n* = 138)
Urinary excretions			
Volume (mL/24 h)	1800 (807)	1782 (858)	1814 (767)
Creatinine (g/24 h)	1.36 (0.51)	1.66 (0.53)	1.11 (0.33) ****
Sodium (mmol/24 h)	174.7 (72.2)	194.3 (76.8)	158.5 (64.1) ****
Potassium (mmol/24 h)	65.1 (24.6)	70.8 (26.0)	60.5 (22.4) ***
Sodium-to-potassium ratio (mmol/mmol)	2.82 (1.07)	2.87 (1.02)	2.77 (1.12)
Dietary estimates			
Sodium intake ^†^ (mg/24 h)	4220 (1745)	4694 (1855)	3828 (1548) ****
Potassium intake ^†^ (mg/24 h)	3303 (1247)	3589 (1321)	3067 (1134) ***
Na/K intake ratio (mg/mg)	1.34 (0.51)	1.37 (0.48)	1.32 (0.53)
Salt intake (g/day)	10.7 (4.4)	11.9 (4.7)	9.7 (3.9) ****

Results are presented as means (SD). *** *p* ≤ 0.001; **** *p* ≤ 0.0001 vs. men. ^†^ Intake values were calculated by multiplying urinary excretion values by 1.05 for Na and by 1.3 for K (see Methods).

**Table 3:** Sodium intake, potassium intake and sodium-to-potassium ratio in single 24-hour collections for individuals by MedDietScore quartiles.

MedDietScore Quartiles	Sodium Intake (mg Per Day)	Potassium Intake (mg Per Day)	Sodium-to-Potassium Intake Ratio
1 (≤28)	4079 (1893) 3661–4498	3241 (1268) 2961–3522	1.32 (0.48) 1.21–1.42
2 (>28, ≤31)	4361 (1746) 3931–4790	3303 (1369) 2964–3642	1.42 (0.61) 1.27–1.57
3 (>31, ≤34)	3972 (1565) 3532–4413	3215 (1275) 2856–3573	1.29 (0.44) 1.17–1.41
4 (>34)	4424 (1636) 3954–4894	3465 (995) 3180–3751	1.33 (0.51) 1.19–1.48
*p* by ANOVA	0.453	0.735	0.532

Results are presented as means (SD) and 95% confidence intervals (CI).
